# Natural Killer Cells Reprogram Myeloid-Derived Suppressor Cells to Induce TNF-α Release via NKG2D–Ligand Interaction after Cryo-Thermal Therapy

**DOI:** 10.3390/ijms25105151

**Published:** 2024-05-09

**Authors:** Jiaqi You, Shicheng Wang, Yongxin Zhu, Zelu Zhang, Junjun Wang, Yue Lou, Yichen Yao, Yuankai Hao, Ping Liu

**Affiliations:** School of Biomedical Engineering and Med-X Research Institute, Shanghai Jiao Tong University, Shanghai 200030, China; jiaqiyou@sjtu.edu.cn (J.Y.); shichengwang@sjtu.edu.cn (S.W.); zhuyongxin12@sjtu.edu.cn (Y.Z.); zeluzhang1126@sjtu.edu.cn (Z.Z.); sjtuwangjunjun@sjtu.edu.cn (J.W.); ly_017082910045@sjtu.edu.cn (Y.L.); yichenyao@sjtu.edu.cn (Y.Y.); haoyuankai@sjtu.edu.cn (Y.H.)

**Keywords:** natural killer cells, myeloid-derived suppressor cells, NKG2D, tumor necrosis factor (TNF)-α, tumor necrosis factor-α converting enzyme (TACE)

## Abstract

In our previous studies, a novel cryothermal therapy (CTT) was developed to induce systemic long-term anti-tumor immunity. Natural killer (NK) cells were found to play an important role in CTT-induced long-term immune-mediated tumor control at the late stage after CTT, but the underlying mechanism is unclear. Myeloid-derived suppressor cells (MDSCs) are immature myeloid cells that have potent immunosuppressive effects on T cells and weaken the long-term benefits of immunotherapy. Consequently, overcoming MDSC immunosuppression is essential for maintaining the long-term efficacy of immunotherapy. In this study, we revealed that NK cells considerably diminish MDSC accumulation at the late stage after CTT, boost T cell production, increase T cell activation, and promote MDSC maturation, culminating in Th1-dominant CD4^+^ T cell differentiation and enhancing NK and CD8^+^ T cell cytotoxicity. Additionally, NK cells activate ERK signaling in MDSCs through NKG2D-ligand interaction to increase the activity of tumor necrosis factor (TNF)-α converting enzyme (TACE)-cleaved membrane TNF-α. Furthermore, Increased TACE activity releases more soluble TNF-α from MDSCs to promote MDSC maturation. In our studies, we propose a novel mechanism by which NK cells can overcome MDSC-induced immunosuppression and maintain CTT-induced persistent anti-tumor immunity, providing a prospective therapeutic option to improve the performance of cancer immunotherapy.

## 1. Introduction

Immunotherapy has yielded remarkable clinical outcomes in cancer patients. However, most patients do not benefit from immunotherapy. Immunotherapies such as the PD-1/PD-L1 immune checkpoint blockade, which is widely used in clinical practice, focus on enhancing T cell activity [1]; however, the complexity of the tumor microenvironment (TME) limits the response. The unsatisfactory efficacy of immunotherapy is partially due to the role of immunosuppressive myeloid cells in the suppression of anti-tumor immunity [2,3]. Myeloid-derived suppressor cells (MDSCs) are a community of immature myeloid cells (IMCs) that can potently suppress immune cell effector functions during tumor progression [4]. MDSCs can create and promote an immunosuppressive tumor environment that is associated with the anergy of T and natural killer (NK) cells [5,6]. In addition, high accumulation of MDSCs is associated with poor prognosis in cancer patients [7]. Therefore, it is imperative to modulate myeloid cells, reduce their accumulation, and inhibit their recruitment or reprogramming of the immunosuppressive function, thereby promoting anti-tumor immunity and improving the therapeutic efficacy of tumor immunotherapy [4,8], ultimately leading to survival benefits.

We developed cryo-thermal therapy (CTT) by combining cooling and radiofrequency ablation for the treatment of solid tumors. CTT markedly reduces the accumulation of MDSCs and promotes the maturation and differentiation of MDSCs into DCs and M1 macrophages, leading to Th1-dominant differentiation of CD4^+^ T cells [9,10,11]. Th1-dominant CD4^+^ T cells generate IFN-γ to maintain the maturation of MDSCs and thus mediate CTT-induced long-term anti-tumor immunity [11]. Moreover, in our previous studies, we found that NK cells also maintain CTT-induced long-term anti-tumor immunity [2]. However, how NK cells mediate CTT-induced long-term anti-tumor immunity has not been addressed.

NK cells are vital, innate cytotoxic lymphocytes that are capable of modulating both the innate and adaptive immune responses to defend the host against infection and malignancy [12]. The activation and function of NK cells must be accurately steered by the relative balance of activating and inhibitory receptors [12,13]. NK cells and myeloid cells are two cell types of the innate immune system that shape the TME and can initiate anti-tumor immune responses. Crosstalk between NK cells and myeloid cells has been shown to play a powerful immunomodulatory role; for example, activated NK cells promote DC maturation to initiate more effective innate and adaptive immunity and facilitate M1 macrophage polarization via IFN-γ [14,15,16]. Activated NK cells can also secrete cytokines such as tumor necrosis factor (TNF)-α to promote myeloid cell maturation [17,18]. Nevertheless, the interactions between NK cells and myeloid cells that affect immune responses are just emerging, and the role of NK cells in durable anti-tumor immunity has also been underexplored.

In this study, to elucidate the role of NK cells in maintaining CTT-induced long-term anti-tumor immunity, a B16F10 melanoma model was established. And NK cells were depleted at the late stage after CTT in vivo, resulting in a marked decrease in mice survival. In vivo studies have shown that NK cells significantly reduce the accumulation of MDSCs, increase T cell production, and promote MDSC maturation and T cell activation. Furthermore, 3′ RNA-seq and in vitro studies have shown that NK cells reverse the immunosuppression of MDSCs and facilitate MDSC maturation, resulting in Th1 CD4^+^ T cell differentiation and enhanced cytotoxicity of NK and CD8^+^ T cells. Moreover, we revealed that NK cells activate ERK signaling in MDSCs via NKG2D-ligand interaction, which enhances the activity of TNF-α converting enzyme (TACE)-cleaved membrane TNF-α. Furthermore, increased TACE activity results in the release of more soluble TNF-α from MDSCs, thus promoting MDSC maturation at the late stage after CTT. Overall, we highlighted a novel mechanism based on the NKG2D–ligand interaction whereby NK cells reprogram the immunosuppressive properties of MDSCs and promote the maturation of MDSCs.

## 2. Results

### 2.1. NK Cells at the Late Stage after CTT Played an Important Role in Maintaining the Long-Term Survival of Mice

**Figure 1:**
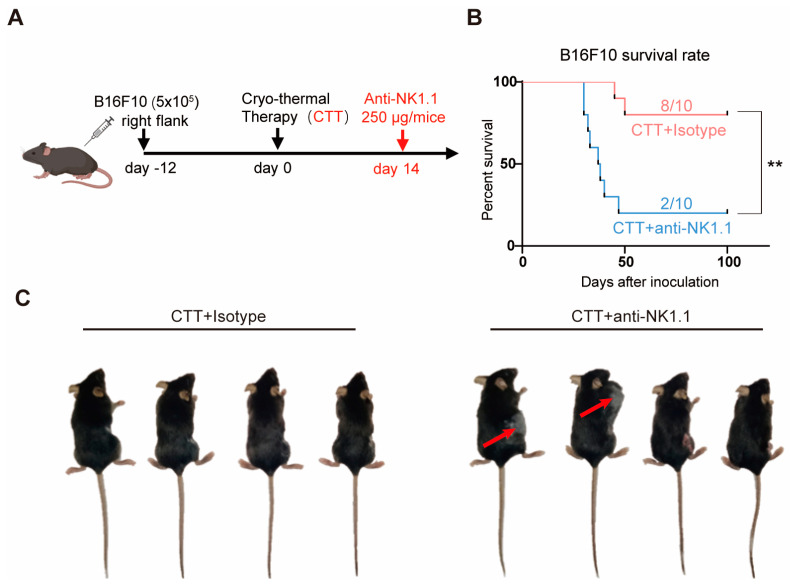
NK cells at the late stage after CTT maintained the long-term survival of mice. (**A**) Experimental design protocol. (**B**) The Kaplan–Meier survival curve of B16F10-tumor-bearing mice after single CTT or CTT with NK cell depletion treatment (n = 10 per group). The log-rank test was used for data analysis. (**C**) Photographic images of tumor metastases after CTT with NK cell depletion (n = 4 per group). ** *p* < 0.01.

Our previous study revealed that NK cell depletion at the late stage after CTT leads to the formation of numerous tumor nodules in the lung, indicating that NK cells at the late stage after CTT are critical for CTT-induced long-term anti-tumor immunity [2]. However, the role of NK cells in mediating durable anti-tumor immunity at the late stage after CTT remains unclear. In this study, to understand the role of NK cells in long-term anti-tumor immunity at the late stage after CTT, mice bearing B16F10 subcutaneous tumors were treated with CTT on day 12 after inoculation, and 250 μg of anti-NK1.1 or isotype mAb was injected intraperitoneally (i.p.) beginning on day 14 after CTT (Figure 1A). The results showed that the survival rate of mice was significantly decreased after CTT with NK cell depletion compared to those without the antibody treatment (Figure 1B). Furthermore, half of the mice (50%) treated with the anti-NK1.1 mAb metastasized within 14 days after CTT, while none of the mice gradually experienced metastases after CTT (Figure 1C). These data suggested that NK cells at the late stage (14 d) after CTT play an important role in maintaining long-term anti-tumor immunity, leading to a significant increase in the survival rate of mice after treatment.

### 2.2. Activated NK Cells Promoted the Maturation of Myeloid Cells and the Activation of T Cells as Well as Regulating T Cell Differentiation at the Late Stage after CTT

**Figure 2:**
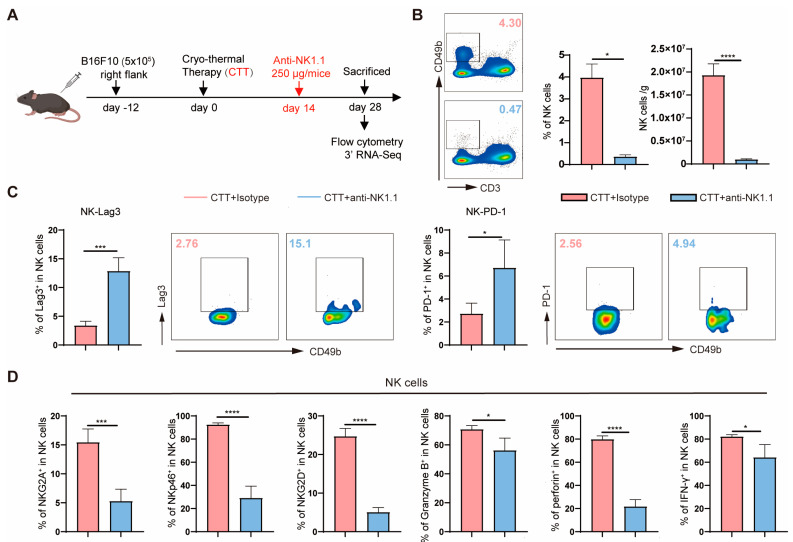
NK cells were activated by CTT. (**A**) Experimental design protocol. (**B**) The percentage and absolute number of splenic NK cells. (**C**) The expression of Lag3 and PD-1 on NK cells educated by CTT and naive NK cells in the spleen. (**D**) The expression of NKG2A, NKp46, NKG2D, Granzyme B, perforin, and IFN-γ in NK cells educated by CTT and naive NK cells in the spleen. All data are shown as mean  ±  SD. n = 4 for each group. * *p* < 0.05, *** *p* < 0.001, **** *p* < 0.0001. Data for graphs were calculated by using two-sided Student’s *T*-test.

To investigate the role of NK cells at the late stage (14 d) after CTT in durable T cell anti-tumor immunity, the efficiency of NK cell depletion was first determined using serial blood draws collected during the two weeks in mice after CTT with NK cell depletion (Figure 2A). The results showed that anti-NK1.1 mAb treatment reduced the proportion of NK cells in the blood after CTT from 7.24% to 0.55% within two days after NK cell depletion (Figure S2A). The proportion of naive NK cells, a population of NK cells that had not been educated by CTT, gradually increased from day 2 after NK cell depletion to approximately 2.83% on day 14 (Figure S2A). Furthermore, compared with those in the CTT group without NK cell depletion, the proportion and absolute number of NK cells in the spleen and blood were decreased after CTT with NK cell depletion (Figure 2B and Figure S2B). In addition, the expression of Lag3 and PD-1 on naive NK cells from the spleen and blood was significantly higher than that on NK cells educated by CTT (Figure 2C and Figure S2B), whereas the expression of CTLA4 on naive NK cells and NK cells educated by CTT in the spleen and blood was similar (Figure S2C). Significantly, the expression of the inhibitory receptor NKG2A; the activating receptors NKG2D and NKp46; and the cytotoxic molecules granzyme B, perforin, and IFN-γ in naive splenic and blood NK cells was markedly downregulated compared to that in NK cells educated by CTT (Figure 2D and Figure S2B). These results revealed that a small population of naive NK cells is produced. Naive NK cells are activated to a lower extent and are less cytotoxic than NK cells educated by CTT.

**Figure 3:**
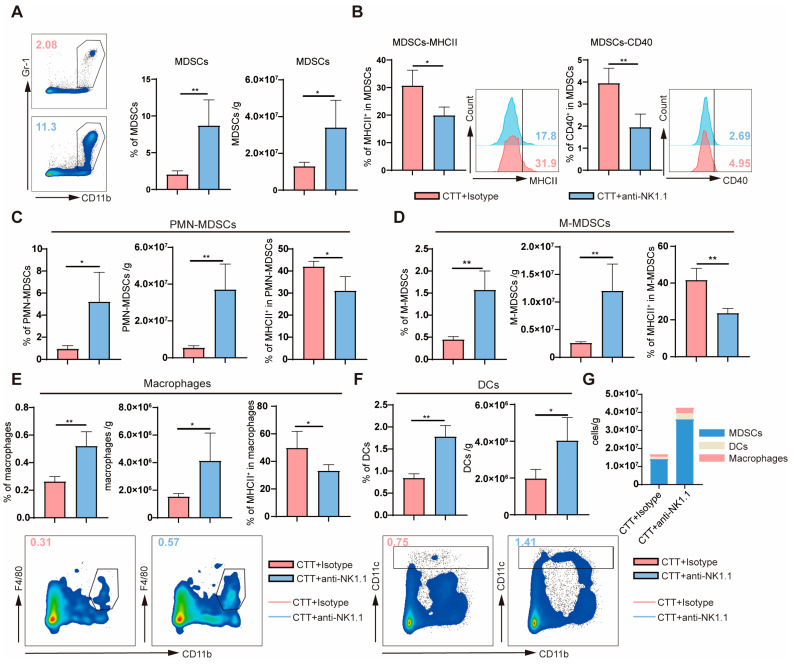
Activated NK cells promoted the maturation of myeloid cells at the late stage after CTT. (**A**) The proportion and absolute number of splenic MDSCs. (**B**) The expression of MHC II and CD40 on splenic MDSCs. (**C**–**E**) The percentage, absolute number, and MHC II expression on PMN-MDSCs (**C**), M-MDSCs (**D**), and macrophages (**E**) in the spleen. (**F**) The percentage and absolute number of splenic DCs. (**G**) The changed absolute number of MDSCs, DCs, and macrophages in two groups. All data were shown as mean  ±  SD. n = 4 for each group. * *p* < 0.05, ** *p* < 0.01. Data for graphs were analyzed using two-sided Student’s *T*-test.

Moreover, myeloid cells and lymphocytes were determined using flow cytometry on day 28 after CTT (Figure 2A). Similarly, compared with those in the CTT group, the proportion of MDSCs in the spleen and blood was significantly increased, and the absolute number of MDSCs was increased after CTT with NK cell depletion (Figure 3A and Figure S3F). MHC II, CD86, and CD40 are involved in the maturation of myeloid cells [19]. The expression of MHC II and CD40 on MDSCs from the spleen and blood after CTT with NK cell depletion was markedly decreased compared with that in CTT-treated mice (Figure 3B and Figure S3F). The expression of CD86 on MDSCs from the spleen and blood after CTT with NK cell depletion was comparable to that in CTT-treated mice (Figure S3A,F). MDSCs can be divided into two types: PMN-MDSCs and M-MDSCs [20,21]. The proportion and absolute number of splenic PMN-MDSCs and M-MDSCs were significantly increased after CTT with NK cell depletion than in CTT-treated mice (Figure 3C,D). Furthermore, compared to that in the CTT group, the expression of MHC II on splenic PMN-MDSCs and M-MDSCs after CTT with NK cell depletion was significantly downregulated (Figure 3C,D). Although the absolute number of PMN-MDSCs and M-MDSCs in the blood was not obviously changed, a significantly increased percentage of PMN-MDSCs and M-MDSCs was observed in the blood after CTT with NK cell depletion (Figure S3G,H). Moreover, the frequency of MHC II^+^ PMN-MDSCs, MHC II^+^ M-MDSCs, and CD86^+^ M-MDSCs in the blood was markedly reduced after CTT with NK cell depletion compared to that in the CTT group (Figure S3G,H). These results indicated that NK cells at the late stage (14 d) after CTT decrease the accumulation of MDSCs and promote MDSC maturation.

Changes in macrophages and DCs were also measured via flow cytometry. The percentage and absolute number of splenic macrophages and DCs in the CTT with NK cell depletion group were markedly higher than those in the CTT group (Figure 3E,F). However, the expression of MHC II on macrophages in the spleen was lower after CTT with NK cell depletion than in CTT-treated mice (Figure 3E). Compared to that in the CTT group, the absolute number of DCs in the blood tended to increase in the CTT with NK cell depletion group, albeit not significantly (Figure S3I). However, the proportion of DCs in the blood was significantly increased after CTT with NK cell depletion (Figure S3I). At the same time, the expression of MHC II, CD86, and CD40 on DCs exhibited no obvious change, and even a declining trend in the blood and spleen (Figure S3E,I). Notably, compared to macrophages and DCs in the spleen, splenic MDSCs were the highest absolute number of all types of myeloid cells in vivo (Figure 3G). And CTT combined with NK cell depletion had a significant impact on the accumulation and maturation of MDSCs. In summary, these results suggested that NK cells at the late stage (14 d) after CTT play a vital role in reducing the accumulation of myeloid cells and promoting the maturation of myeloid cells, particularly MDSCs.

**Figure 4:**
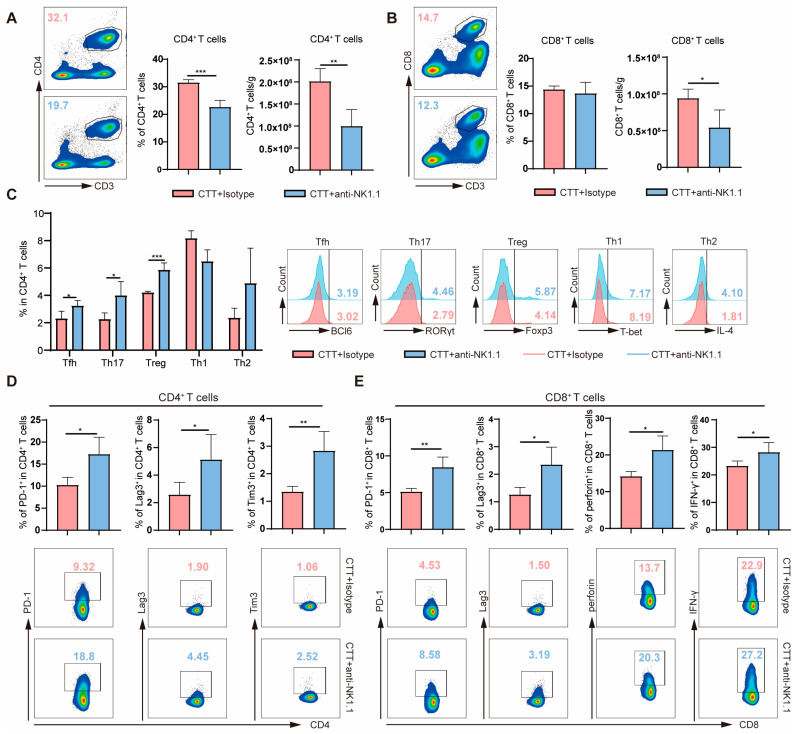
Activated NK cells promoted the activation of T cells and regulated T cell differentiation at the late stage after CTT. (**A**,**B**) The percentage and absolute number of CD4^+^ T cells (**A**) and CD8^+^ T cells (**B**) in the spleen. (**C**) The subsets of splenic CD4^+^ T cells. (**D**) The expression of PD-1, Lag3, and Tim3 on splenic CD4^+^ T cells. (**E**) The expression of PD-1, Lag3, perforin, and IFN-γ in splenic CD8^+^ T cells. All data are shown as mean  ±  SD. n = 4 for each group. * *p* < 0.05, ** *p* < 0.01, *** *p* < 0.001. Data were analyzed using two-sided Student’s *T*-test.

To investigate, comprehensively, the changes in lymphocytes at the late stage (14 d) after CTT with NK cell depletion, the accumulation and function of T cells were analyzed 28 days after treatment using flow cytometry. Compared with those in the CTT group, the percentage of splenic CD8^+^ T cells was not significantly different, but the proportion of CD4^+^ T cells and the absolute number of CD4^+^ and CD8^+^ T cells in the spleen were obviously decreased after CTT with NK cell depletion (Figure 4A,B). Meanwhile, the absolute numbers of CD4^+^ and CD8^+^ T cells in the blood did not differ, but there was a notable increase in the percentage of CD4^+^ and CD8^+^ T cells in the blood after CTT with NK cell depletion (Figure S4D,F). These data suggested that NK cells at the late stage (14 d) after CTT promote the accumulation of T cells in the spleen. Notably, larger subsets of splenic Tfh, Th17, and Treg cells were observed after CTT with NK cell depletion, but the percentages of splenic Th1 and Th2 cells did not obviously change (Figure 4C). Furthermore, the expression of PD-1, Lag3, and Tim3 on splenic CD4^+^ T cells was strongly increased after CTT with NK cell depletion compared with that in CTT-treated mice (Figure 4D). Concurrently, compared to those in the CTT group, the abundance of CD4^+^ T cell subsets in the blood and the expression of Lag3, CTLA4, and PD-1 on CD4^+^ T cells in the blood were not significantly affected after CTT with NK cell depletion (Figure S4D,E). However, the expression of Tim3 on peripheral blood CD4^+^ T cells was markedly increased in the CTT with NK cell depletion group than in the CTT group (Figure S4D). These results indicated that NK cells at the late stage (14 d) after CTT inhibit the differentiation of CD4^+^ T cells towards the Tfh, Th17, and Treg subsets and the expression of inhibitory receptors on CD4^+^ T cells. Moreover, compared to those in CTT-treated mice, although higher perforin and IFN-γ expression in splenic CD8^+^ T cells was observed, the PD-1 and Lag3 expression on splenic CD8^+^ T cells was significantly upregulated after CTT with NK cell depletion (Figure 4E). At the same time, unchanged expression of Tim3, CTLA4, and granzyme B in splenic CD8^+^ T cells was observed after CTT with NK cell depletion (Figure 4E and Figure S4B). However, the expression of Lag3 and CTLA4 on peripheral blood CD8^+^ T cells after CTT with NK cell depletion was significantly increased compared with those in the CTT group (Figure S4F). Overall, these findings suggested that NK cells at the late stage (14 d) after CTT regulate the effector function of CD8^+^ T cells and restrain exhausted CD8^+^ T cells.

In conclusion, these data revealed that NK cells at the late stage (14 d) after CTT reduce the accumulation of myeloid cells, especially MDSCs, and boost the generation of T cells, as well as promoting myeloid cell maturation, T cell activation, and the differentiation of T cells to maintain the durable anti-tumor immunity induced by CTT.

### 2.3. NK Cells Were Essential for Reversing the Immunosuppression of MDSCs and Promoting MDSC Maturation at the Late Stage after CTT

**Figure 5:**
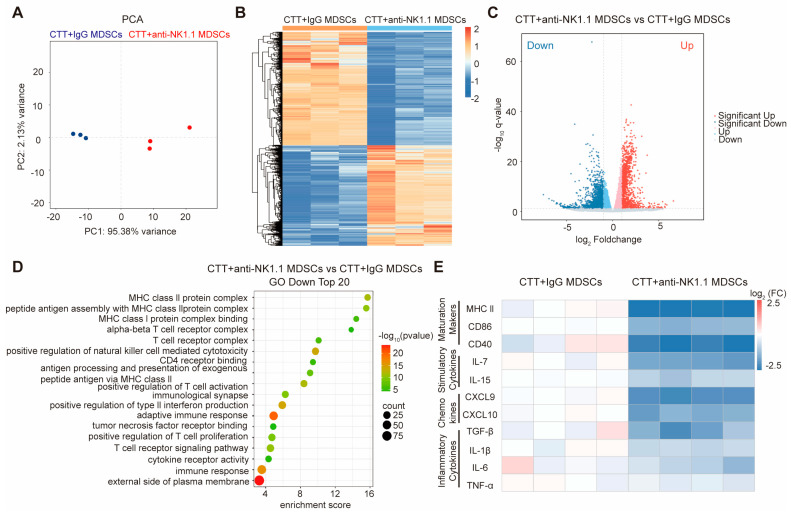
NK cells at the late stage after CTT reversed MDSC immunosuppression and promoted MDSC maturation. (**A**) Principal component analysis (PCA) of the two sample groups. (**B**) Heat map of differentially expressed genes (n = 3 for each group). (**C**) Volcano plot of differentially expressed genes in splenic MDSCs. (**D**) Scatter plot of downregulated GO signaling pathway in splenic MDSCs. (**E**) The mRNA levels of maturation markers, chemokines, stimulatory, and inflammatory cytokines genes in splenic MDSCs were analyzed by qRT-PCR (n = 4 for each group).

The above results revealed that NK cells at the late stage (14 d) after CTT promote the maturation of MDSCs, which are the highest absolute number of myeloid cells, the activation of T cells, and the regulation of T cell differentiation to preserve CTT-triggered durable anti-tumor immunity. At the same time, the maturation of MDSCs effectively induced CD4^+^ Th1 cell differentiation and enhanced the cytotoxic effector function of CD8^+^ T cells after CTT [2,11]. Therefore, we hypothesized that the maturation of MDSCs induced by NK cells at late stage (14 d) after CTT would further affect the function of T cells. To elucidate the mechanism by which NK cells at the late stage (14 d) after CTT regulate MDSC function, splenic MDSCs from mice after CTT or CTT with NK cell depletion were isolated, and 3′ RNA -seq was performed (Figure 2A). The results showed that compared with those in the CTT group, the splenic MDSCs in the CTT with NK cell depletion group experienced enormous changes (Figure 5A,B). Specifically, 2403 genes showed upregulated expression and 2454 genes showed downregulated expression (Figure 5C). Furthermore, gene ontology (GO) enrichment analysis revealed that antigen processing and presentation pathways (including both MHC I and MHC II protein complex binding) were notably downregulated in MDSCs after CTT with NK cell depletion (Figure 5D). Moreover, compared with those in CTT-treated mice, positive regulation of NK cell mediated cytotoxicity, T cell activation, type II interferon production, and T cell proliferation were downregulated in MDSCs after CTT with NK cell depletion (Figure 5D). These data strongly implied that the maturation of MDSCs induced by NK cells at the late stage (14 d) after CTT facilitates NK cell cytotoxicity, T cell activation, type II interferon production, and T cell proliferation.

To further investigate the functional changes in MDSCs after CTT with NK cell depletion, splenic MDSCs from the CTT and CTT with NK cell depletion groups were isolated by using MACS on day 28 after CTT, respectively. The gene expression levels of MDSCs, including those of maturation markers, stimulatory cytokines, chemokines, and inflammatory cytokines, were measured via qRT-PCR. Slightly lower levels of inflammatory cytokines, such as TGF-β, IL-1β, IL-6, and TNF-α were observed in MDSCs after CTT with NK cell depletion than in those from the CTT group (Figure 5E). The expression of maturation markers (MHC II, CD86, and CD40), stimulatory cytokines (IL-7 and IL-15), and chemokines (CXCL9 and CXCL10) in MDSCs was significantly decreased after CTT with NK cell depletion compared to those in the CTT group (Figure 5E). These data verified that NK cells at the late stage (14 d) after CTT are critical for reversing the immunosuppression of MDSCs, as well as promoting the phenotypic and functional maturation of MDSCs.

### 2.4. NK Cells Reversed the Immunosuppression of MDSCs and Promoted MDSC Maturation at the Late Stage after CTT, Which Plays a Critical Role in CTT-Triggered Systemic Anti-Tumor Immunity

**Figure 6:**
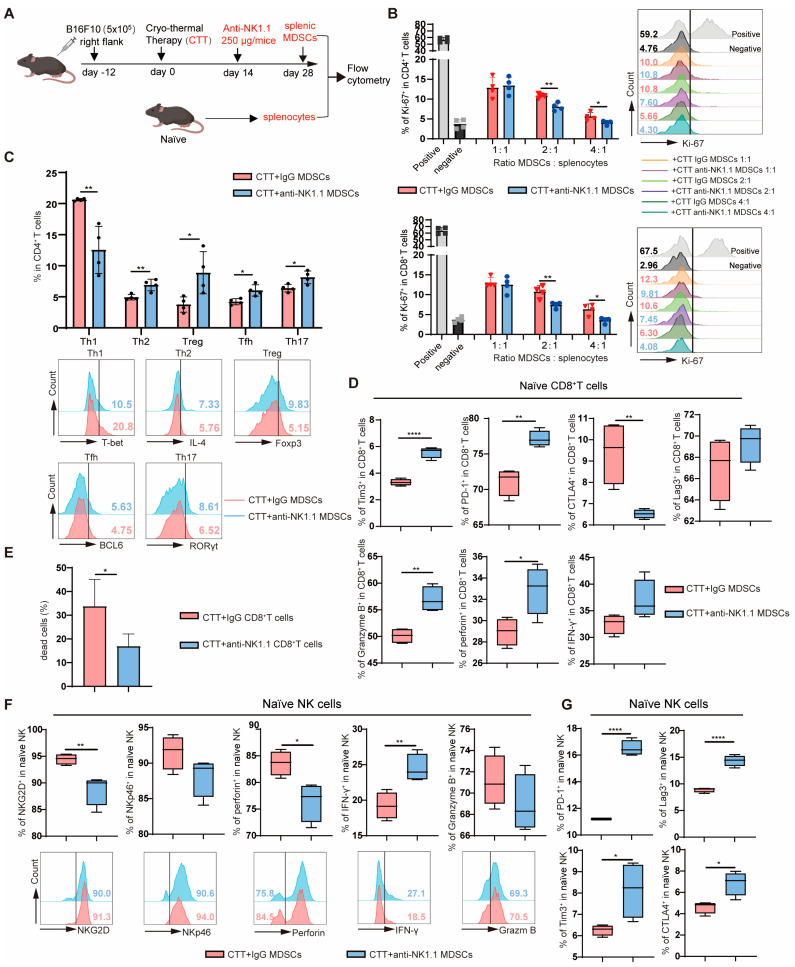
NK cells orchestrated systemic anti-tumor immunity by reversing the immunosuppression of MDSCs and promoting maturation of MDSCs at the late stage after CTT. (**A**) Scheme of in vitro experiment design: splenic MDSCs isolated from CTT and CTT with NK cell depletion groups on day 28 after CTT and cocultured with splenocytes from naive mice. (**B**) MDSCs were cocultured with naive splenocytes for 72 h to detect the expression of Ki-67 in CD4^+^ T cells and CD8^+^ T cells at ratios of 1:1, 2:1, and 4:1; naive splenocytes were cocultured with or without anti-CD3 antibody (1 μg/mL) served as positive or negative groups. (**C**,**D**) MDSCs were cocultured with naive splenocytes for 24 h; then, the subsets of CD4^+^ T cells (**C**), inhibitory molecules (CTLA4, Tim3, PD-1, Lag3), and cytotoxic molecules (granzyme B, IFN-γ, perforin) in CD8^+^ T cells (**D**) were measured via flow cytometry. (**E**) The tumor killing capacity of CD8^+^ T cells from the CTT and CTT with NK cell depletion groups. Splenic CD8^+^ T cells isolated via MACS were cocultured with calcein-AM pre-labeled B16F10 cells at 16:1 for 6 h; then, the fluorescence of supernatant was analyzed via a microplate reader. (**F**,**G**) MDSCs were cocultured with naive splenocytes for 24 h; then, the expression of NKG2D, NKp46, perforin, IFN-γ, Granzyme B (**F**), CTLA4, Tim3, PD-1, and Lag3 (**G**) of naive NK cells was detected via flow cytometry. All data are shown as mean  ±  SD (n = 4 for each group). * *p* < 0.05, ** *p* < 0.01, **** *p* < 0.0001. Data were analyzed using two-sided Student’s *T*-test.

To further assess the effect of MDSCs on T cells after CTT with NK cell depletion, splenic MDSCs were isolated via MACS and cocultured with naive splenocytes at ratios of 1:1, 2:1, and 4:1 for 72 h (Figure 6A). Cell proliferation was assessed via flow cytometry using an antibody against the proliferation-associated nuclear antigen Ki-67. At ratios of 2:1 and 4:1, the frequencies of Ki-67^+^ CD4^+^ T cells and Ki-67^+^ CD8^+^ T cells were observably decreased after coculture with MDSCs from the CTT with NK cell depletion group (Figure 6B). These results implied that NK cells reverse the immunosuppression of MDSCs and promote MDSC maturation, resulting in the promotion of T cell proliferation at the late stage (14 d) after CTT. In addition, to investigate the effect of MDSCs on CD4^+^ T cell differentiation, cytotoxic T lymphocytes (CTLs) and NK cell activation, splenic MDSCs collected from two groups were cocultured with naive splenocytes for 24 h (Figure 6A). These results showed that MDSCs from the CTT with NK cell depletion group suppressed Th1 differentiation of CD4^+^ T cells, but promoted Th2, Treg, Tfh, and Th17 differentiation in comparison to those cocultured with MDSCs from the CTT group (Figure 6C). These data suggested that MDSCs educated by NK cells are critical for facilitating CD4^+^ Th1 cell differentiation and inhibiting the differentiation of CD4^+^ T cells towards Th2, Treg, Tfh, and Th17 cells after CTT.

Moreover, the expression of Tim3 and PD-1 on CD8^+^ T cells was significantly increased when CD8^+^ T cells were cocultured with MDSCs from the CTT with NK cell depletion group, although the expression level of CTLA4 on CD8^+^ T cells was significantly decreased (Figure 6D). Interestingly, when CD8^+^ T cells were cocultured with MDSCs after CTT with NK cell depletion, the expression of granzyme B and perforin in CD8^+^ T cells was notably increased (Figure 6D), which was in accordance with the in vivo results (Figure 3E). Thus, to further assess the cytotoxicity of CD8^+^ T cells, the ability of CD8^+^ T cells to kill targeted tumor cells was studied. Splenic CD8^+^ T cells were isolated via MACS and cocultured with calcein-AM-labeled B16F10 murine melanoma cells to detect calcein fluorescence in the supernatant. The results showed that CD8^+^ T cells after CTT with NK cell depletion were less cytotoxic to B16F10 cells than were those from the CTT group (Figure 6E). These data implied that NK cells at the late stage (14 d) after CTT reverse the immunosuppression of MDSCs and promote MDSC maturation, resulting in the promotion of CD8^+^ T cell cytotoxicity. At the same time, compared with those cocultured with CTT MDSCs, NK cells cocultured with MDSCs from the CTT with NK cell depletion group exhibited significantly lower expression levels of NKG2D and perforin, although IFN-γ expression was slightly upregulated (Figure 6F). The expression of inhibitory molecules (PD-1, Lag3, Tim3, and CTLA4) on naive NK cells was markedly increased after coincubation with MDSCs from CTT with NK cell depletion group than after coculture with MDSCs after CTT (Figure 6G). These data suggested that NK cells at the late stage (14 d) after CTT reverse the immunosuppression of MDSCs and facilitate MDSC maturation, thereby promoting the activation and cytotoxicity of naive NK cells.

In summary, these data indicated that NK cells at the late stage (14 d) after CTT reverse the immunosuppression of MDSCs and promote their maturation, leading to increased T cell proliferation and CD4^+^ Th1 cell differentiation. They also enhance the cytotoxicity of CD8^+^ T and NK cells, thereby orchestrating systemic anti-tumor immunity.

### 2.5. NKG2D–Ligand Interaction Promoted MDSC Maturation by Increasing TACE Activity in MDSCs through ERK Signaling, Which, in Turn, Increased the Release of Soluble TNF-α from MDSCs

**Figure 7:**
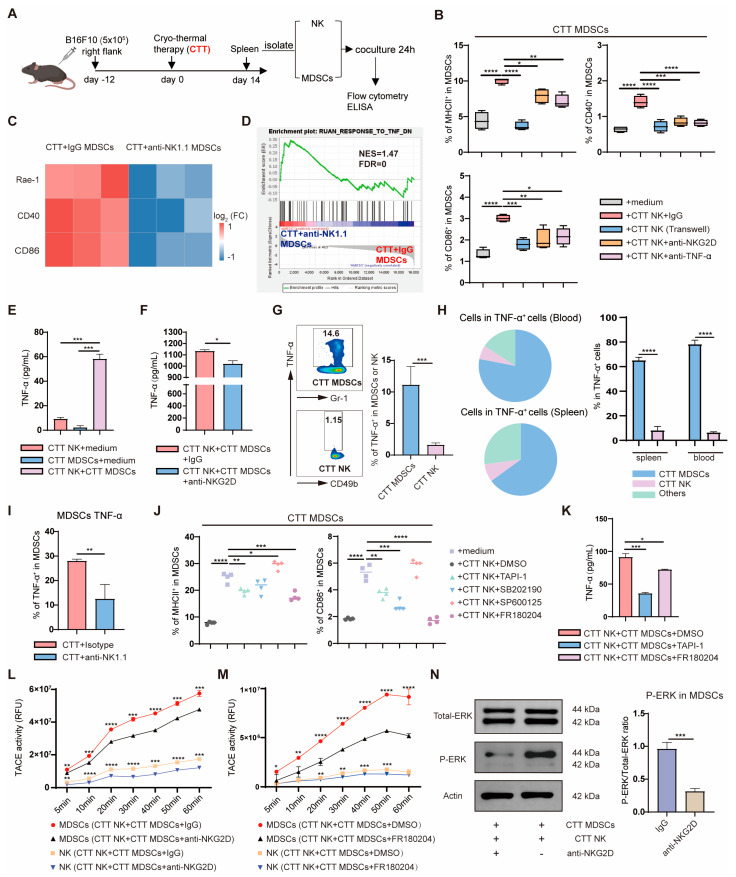
NKG2D–ligand interaction increased the release of soluble TNF-α from MDSCs, thus promoting MDSC maturation. (**A**) Scheme of in vitro experiment design: splenic NK cells and MDSCs were isolated on day 14 after CTT and cocultured for 24 h. (**B**) MDSCs and NK cells were cocultured with the addition of anti-NKG2D (15 μg/mL) and anti-TNF-α (10 μg/mL) antibodies or were separated by a 0.4 μm transwell chamber for 24 h. The expression of MHC II, CD86, and CD40 was detected via flow cytometry (n = 4 for each group). (**C**) Fragments per kilobase million (FPKM) of Rae-1, CD40, and CD86 (n = 3 for each group). (**D**) Individual GSEA enrichment plot for the process that responds to reduced TNF-α. (**E**) The levels of TNF-α in the supernatant from NK cells, MDSCs, and coculture system (containing NK cells and MDSCs) were determined via ELISA. (**F**) The concentration of TNF-α released in the supernatant of the coculture system (containing NK cells and MDSCs) in the presence or absence of anti-NKG2D (15 μg/mL) antibody were determined via ELISA. (**G**) The TNF-α expressive capacity of MDSCs and NK cells after CTT. (**H**) The compositions of TNF-α^+^ cells in the spleen and blood were detected via flow cytometry. (**I**) Mice were treated with anti-NK1.1 antibody on day 14 after CTT, and the expression of TNF-α in MDSCs was analyzed on day 28 after CTT via flow cytometry (n = 4 for each group). (**J**) TAPI-1 (TACE inhibitor), SB202190 (p38 MAPK inhibitor), SP600125 (JNK inhibitor), and FR180204 (ERK inhibitor) were added to the coculture system of MDSCs and NK cells, and the expression of MHC II, CD86, and CD40 was detected via flow cytometry (n = 4 for each group). (**K**) TNF-α released in the supernatant of coculture system (containing NK cells and MDSCs) with TAPI-1 (TACE inhibitor) and FR180204 (ERK inhibitor) was determined via ELISA. (**L**,**M**) MDSCs and NK cells were cocultured and supplied with anti-NKG2D (15 μg/mL) antibody (**L**) and FR180204 (ERK inhibitor, 10 μM) (**M**) for 24 h, and then MDSCs and NK cells from the coculture system were isolated via MACS for TACE activity detection, respectively (n = 3 for each group). (**N**) Anti-NKG2D (15 μg/mL) antibody was added to the coculture system (containing NK cells and MDSCs) for 3 h; then, MDSCs were isolated via MACS from the coculture system, ERK signaling was analyzed via Western blot, and the samples derived from the same experiment and blot were processed in parallel (n = 3 for each group). All data are shown as mean  ±  SD. * *p* < 0.05, ** *p* < 0.01, *** *p* < 0.001, **** *p* < 0.0001. For two groups, data were analyzed using two-sided Student’s *T*-test; for multiple groups, data were analyzed using one-way ANOVA.

The aforementioned results indicated that NK cells at the late stage (14 d) after CTT facilitate MDSC maturation. Furthermore, to elucidate the mechanism by which NK cells promote the maturation of MDSCs, splenic NK cells and MDSCs were isolated on day 14 after CTT via MACS. Cells were cocultured or separated via a transwell chamber for 24 h, and the expression levels of maturation markers (MHC II, CD86 and CD40) on MDSCs were assessed via flow cytometry (Figure 7A). NK cells educated by CTT strongly promoted MDSC maturation when directly cocultured with MDSCs together compared to when MDSCs were cultured in vitro alone, as evidenced by a significant increase in the expression of MHC II, CD86, and CD40 (Figure 7B). However, the expression of MHC II, CD86, and CD40 on MDSCs that were separated from NK cells was dramatically decreased (Figure 7B). These data implied that NK cells educated by CTT promote the maturation of MDSCs in a cell-to-cell contact-dependent manner. Thus, the ligand expression of NK cells on MDSCs was analyzed using 3′ RNA-seq data. The results showed that the expression of Rae1, CD40, and CD86 on MDSCs after CTT with NK cell depletion was downregulated compared to that on MDSCs after CTT (Figure 7C). Furthermore, the response of MDSCs to TNF-α was downregulated after CTT with NK cell depletion (Figure 7D). In addition, cytokine receptor activity and TNF receptor binding in MDSCs after CTT with NK cell depletion were significantly reduced (Figure 5D). Moreover, TNF-α plays an important role in promoting myeloid cell maturation [18,22]. Therefore, anti-NKG2D, anti-CD40L, anti-CD86, and anti-TNF-α antibodies were added to the coculture system containing NK cells and MDSCs on day 14 after CTT for 24 h. The results showed that the addition of anti-NKG2D and anti-TNF-α antibodies significantly downregulated the expression levels of MHC II, CD86, and CD40 on MDSCs after they were cocultured with NK cells (Figure 7B). However, the addition of other blocking reagents specific for CD40L and CD86 to the cocultures did not affect the expression of these molecules on MDSCs (Figure S5A). Similarly, it was found that in vitro blockade of NKG2D and TNF-α in 4T1 model also inhibited the maturation of MDSCs (Figure S5C). These data suggested that NKG2D and TNF-α play key roles in the maturation of MDSCs.

The means by which NKG2D and TNF-α affect the maturation of MDSCs should be addressed. NKG2D–ligand interaction significantly increases the release of TNF-α [22]. Therefore, we speculated that TNF-α release is affected by the NKG2D–ligand interaction between NK cells and MDSCs, which facilitates MDSC maturation. Then, TNF-α in the coculture supernatant was detected via ELISA. The results showed that the level of TNF-α in the supernatant after coincubation of NK cells and MDSCs after CTT was significantly increased compared to NK cell or MDSC culture alone (Figure 7E). These data implied that the interaction between NK cells and MDSCs after CTT significantly promotes the release of TNF-α. Apart from this, the amount of TNF-α secreted into the coculture supernatant was significantly reduced when the anti-NKG2D antibody was added to the coculture system containing NK cells and MDSCs (Figure 7F). These data suggested that the NKG2D–ligand interaction between NK cells and MDSCs enhances the release of TNF-α, leading to MDSC maturation.

Furthermore, to identify the predominant source of TNF-α produced by NK cells or MDSCs, the expression levels of TNF-α in MDSCs and NK cells after CTT were determined by using flow cytometry. The total capacity of MDSCs to produce TNF-α was markedly higher than that of NK cells after CTT (Figure 7G). Additionally, TNF-α^+^ cells in the spleen and blood after CTT were also analyzed using flow cytometry. The results showed that the majority of TNF-α^+^ cells were MDSCs, which accounted for approximately 65% of the TNF-α^+^ cells in the spleen and almost 78% of the TNF-α^+^ cells in the blood (Figure 7H). However, only 8% of the splenic and 6% of the peripheral blood TNF-α^+^ cells were NK cells (Figure 7H). Moreover, the ability of MDSCs to produce TNF-α was also inhibited after CTT with NK cell depletion compared to that of MDSCs after CTT in vivo (Figure 7I). These results suggested that NK cells markedly promote TNF-α production in MDSCs after CTT. All these data demonstrated that the NKG2D–ligand interaction between NK cells and MDSCs mainly enhances TNF-α release from MDSCs, leading to MDSC maturation after CTT.

TNF-α is originally produced and expressed as a transmembrane protein on the cell surface, and membrane TNF-α must be cleaved by TACE to produce soluble TNF-α [23]. Elevated TACE activity can accelerate the release of soluble TNF-α from membrane TNF-α [22]. According to the aforementioned finding that the NKG2D–ligand interaction enhances TNF-α release from MDSCs following coculture of NK cells and MDSCs after CTT, resulting in the maturation of MDSCs (Figure 7B,E), we then surmised that the NKG2D–ligand interaction increases soluble TNF-α release from MDSCs by enhancing TACE activity to cleave membrane TNF-α, leading to MDSC maturation. To verify whether the maturation of MDSCs is caused by TACE-mediated TNF-α release, splenic NK cells and MDSCs from CTT-treated mice were cocultured with the TAPI-1 (TACE inhibitor) for 24 h in vitro. The maturation of MDSCs and the amount of TNF-α in the supernatant were measured via flow cytometry and ELISA, respectively. The results showed that the expression of MHC II and CD86 on MDSCs was significantly downregulated after coculture with TAPI-1 (TACE inhibitor) (Figure 7J). The level of TNF-α in the supernatant was markedly decreased after the addition of TAPI-1 (TACE inhibitor) (Figure 7K). These data suggested that TACE is required for the release of TNF-α from MDSCs following coculture with NK cells. Then, whether TACE activity is affected by the NKG2D–ligand interaction was determined. And TACE activity in MDSCs and NK cells from the coculture system, including NK cells and MDSCs after CTT, was measured when NKG2D was blocked. NKG2D blockade led to a more significant decrease in TACE activity in MDSCs than in NK cells after CTT (Figure 7L). Moreover, TACE activity in NK cells was very low compared to that in MDSCs. These data implied that the NKG2D–ligand interaction mainly enhances TACE activity in MDSCs to release soluble TNF-α, which promotes MDSC maturation after CTT.

MAPK signaling pathway is required to deregulate TACE inhibition and to enhance TACE activity to promote TNF-α release [24,25,26,27,28]. To further investigate the role of MAPK signaling in the promotion of MDSC maturation, a MAPK inhibitor (containing a p38 MAPK, ERK and JNK inhibitor) was used to suppress MAPK signaling transduction in a coculture system of splenic NK cells and MDSCs 14 days after CTT for 24 h. The expression of maturation markers (MHC II, CD86, and CD40) on MDSCs was analyzed using flow cytometry. As shown in Figure 7J, the expression levels of MHC II and CD86 on MDSCs were significantly decreased by the addition of FR180204 (ERK inhibitor) but not by the addition of SB202190 (p38 MAPK inhibitor) and SP600125 (JNK inhibitor). Then, the level of TNF-α in the coculture supernatant and the TACE activity of MDSCs and NK cells were measured after NK cells and MDSCs from the CTT group were cocultured with FR180204 (ERK inhibitor) for 24 h. The results showed that the level of TNF-α in the supernatant significantly decreased after ERK signaling was inhibited (Figure 7K). Furthermore, TACE activity in MDSCs was markedly downregulated when ERK signaling was blocked (Figure 7M). In addition, the phosphorylation level of ERK signaling in MDSCs from the coculture system, including NK cells and MDSCs after CTT, was measured when anti-NKG2D antibody was added. The results showed that the phosphorylation level of ERK was significantly inhibited after NKG2D blockade (Figure 7N). These data suggested that activated ERK signaling in MDSCs through NKG2D–ligand interaction enhances TACE activity, which promotes soluble TNF-α production and facilitates MDSC maturation after CTT.

Taken together, these findings suggested that NK cells at the late stage (14 d) after CTT depend on the NKG2D–ligand interaction to upregulate TACE activity in MDSCs via ERK signaling, thereby increasing soluble TNF-α release from MDSCs and promoting MDSC maturation.

## 3. Discussion

In our previous studies, we demonstrated that Th1 cells are the dominant in subset of CD4^+^ T cells at the late stage after CTT and mediate long-term anti-tumor immunity [11]. However, NK cell depletion at the late stage after CTT results in the growth of numerous tumor nodules in the lung, implying that NK cells also play a role in CTT-triggered long-term anti-tumor immunity to inhibit pulmonary metastases [2]. But the mechanism by which NK cells preserve durable anti-tumor immunity at the late stage after CTT is still unknown. In this study, the role of NK cells in CTT-induced long-term anti-tumor immunity was further addressed. We revealed that at the late stage after CTT, NK cells activate ERK signaling of MDSCs through the NKG2D–ligand interaction, which, in turn, upregulates TACE activity to cleave membrane TNF-α. Upregulated TACE activity subsequently increases soluble TNF-α release and promotes MDSC maturation. Furthermore, mature MDSCs lead to effective CD4^+^ Th1-dominant anti-tumor immunity and result in a significantly increased survival rate in mice.

NK cells are lymphocytes of the innate immune system and constitute the first line of host defense. NK cells can directly kill tumor cells and interact with other immune cells, such as DCs and CD4^+^ T cells, to promote the maturation of DCs or the differentiation of Th1 cells [2,29]. However, in patients with advanced cancer, the function of NK cells is often impaired [30]. It has been discovered that NK cells can regulate long-term immune responses and produce memory in the chemical hapten-induced contact hypersensitivity (CHS), human immunodeficiency virus-1 (HIV-1), influenza viruses, mouse cytomegalovirus (MCMV), and vesicular stomatitis virus (VSV) models [31,32,33,34,35]. However, the role of NK cells in long-term anti-tumor immunity has not been reported in tumor models. Therefore, in this study, investigating the role of NK cells in persistent anti-tumor immunity could further our understanding of the mechanisms of persistent anti-tumor immunity induced by CTT. More crucially, we revealed the interplay between NK cells and MDSCs, which provides new insight into the therapeutic effects of CTT.

NKG2D is a dominant NK-cell-activating receptor whose ligand binding induces granule release and cytokine production in NK cells. NKG2D–ligand interaction can kill MDSCs and enhance TACE activity to release soluble TNF-α from NK cells [2,22]. In addition, the NKG2D–ligand interaction can stimulate effector functions in NK cells, eliminate MICA/B-expressing tumor cells through NK cells, and reprogram tumor-associated macrophages (TAMs) into proinflammatory macrophages [36,37]. Our present study demonstrated that the NKG2D–ligand interaction enhances TACE activity to release soluble TNF-α, leading to MDSC maturation. Furthermore, mature MDSCs effectively promote CD4^+^ Th1 differentiation to sustain CTT-triggered persistent anti-tumor immunity. To our knowledge, the underlying mechanism has not been reported in other studies. Moreover, our studies also suggested that enhancing the NKG2D–ligand interaction via NK cells and MDSCs and promoting the release of soluble TNF-α could be used to improve NK cell-based immunotherapies.

Previous studies discovered that NK cells at the early stage after CTT could kill MDSCs via the NKG2D–ligand interaction and promote the maturation of MDSCs obtained from tumor-bearing mice via IFN-γ [2]. But in the present study, MDSCs were obtained from CTT-treated mice at the late stage after treatment, which were fully mature phenotypes compared to MDSCs obtained from tumor-bearing mice in our previous experimental systems. We found that NK cells induced TNF-α release from MDSCs via the NKG2D–ligand interaction, leading to maintenance of the maturation of MDSCs, while IFN-γ had a weaker effect on maintaining the maturation of MDSCs at the late stage after treatment. Mature myeloid cells with high expression of MHC II and CD86 become antigen-presenting cells (APCs) that promote the polarization of Th1 cells [38]. The CD40-CD40L interaction is a crucial costimulatory signal in T cell activation [39]. The CD40-CD40L interaction between myeloid cells and T cells can facilitate the production of IL-12 in myeloid cells to trigger Th1 immunity [40,41]. Mature MDSCs can induce the differentiation of CD4^+^ Th1 cells to maintain durable anti-tumor immunity [2,8]. Our previous studies revealed that NK cells could promote the maturation of MDSCs from tumor-bearing mice through NK cell-derived IFN-γ, as evidenced by significantly increased expression levels of MHC II and CD86 [2]. However, in the present study, we found that anti-TNF-α treatment significantly decreased the expression of MHC II, CD86, and CD40 on MDSCs from CTT-treated mice when cocultured with NK cells. However, only the expression of CD40 on MDSCs from CTT-treated mice was markedly downregulated when anti-IFN-γ antibody was added to the coculture system containing NK cells and MDSCs educated by CTT (Figure S5B). And the expression of MHC II and CD86 remained unchanged (Figure S5B). These data indicated that IFN-γ promote the expression of MHC II and CD86 during the maturation of MDSCs derived from untreated tumor-bearing mice. But in MDSCs educated by CTT, IFN-γ slightly affected the expression of MHC II and CD86. However, TNF-α could play a more prominent role in sustaining the expression of MHC II and CD86 on mature MDSCs educated by CTT. In this study, the role of IFN-γ and TNF-α on MDSCs at different mature states was preliminary. Further, elucidating the various discrepancies and comparing the phenotypic differences in MDSCs after CTT with IFN-γ or TNF-α depletion, as well as their respective roles in regulating cellular functions, are worthy courses of investigation in the near future.

Importantly, TNF-α and IFN-γ treatment could increase the expression of CD40 on MDSCs educated by CTT when cocultured with NK cells. Therefore, our studies suggested that at the late stage after CTT, TNF-α could sustain the expression of MHC II and CD86 on mature MDSCs educated by CTT. Moreover, TNF-α and IFN-γ could induce high levels of CD40 on mature MDSCs through interacting with CD40L on T cells after CTT to promote Th1-dominant CD4^+^ T cell differentiation and orchestrate CTT-induced long-term anti-tumor immunity. Both MDSCs and NK cells can produce TNF-α for immunomodulation [17,22,42]. In the present study, we discovered that the in vitro culture of either MDSCs or NK cells alone produced a small amount of TNF-α. However, coculture of the two types of cells significantly enhanced the ability of MDSCs to produce TNF-α, and more soluble TNF-α was released by MDSCs. Furthermore, we showed that this is mainly because cell-to-cell contact between NK cells and MDSCs significantly increases TACE activity in MDSCs rather than that in NK cells, allowing MDSCs to release more soluble TNF-α.

Interestingly, in our study, CTT combined with NK cell depletion treatment decreased the expression of IFN-γ and perforin in splenic CD8^+^ T cells but increased PD-1, Lag3, and Tim3 expression in splenic CD8^+^ T cells in vivo, which was consistent with the finding of the in vitro study. PD-1^+^ Tim3^+^ CD8^+^ T cells retain the potential to produce IFN-γ but lack cytotoxicity in ovarian cancer [43]. In addition, a killing assay showed that CD8^+^ T cells decreased the tumor killing capacity after CTT with NK cell depletion, which indicated that NK cells at the late stage (14 d) promote the cytotoxicity of CD8^+^ T cells. However, the exact mechanism through which NK cells regulate the function of CD8^+^ T cells after CTT requires further investigation.

After CTT with NK cell depletion, a population of naive NK cells that were not educated by CTT was observed to be less cytotoxic and more inhibitory than NK cells educated by CTT. This could be because the proportion and absolute number of immature MDSCs increased after CTT combined with NK cell depletion, which impaired the effector functions of naive NK cells. MDSCs can cause NK cell dysfunction [44,45]. MDSCs induce NK cell inactivation, decrease the expression of NK-cell-activating receptors (e.g., NKG2D), interfere with NK cell cytotoxicity, and simultaneously reduce IFN-γ production to impair anti-tumor immunity [46,47]. Moreover, the frequency of MDSCs was found to be inversely correlated with the expression of NK cell-activated receptors [48].

In our previous studies, CTT was shown to induce CD4^+^ Th1-dominant anti-tumor immunity to sustain the long-term survival of mice [11]. Th1-dominant CD4^+^ T cells can activate NK cells and increase NK cell cytotoxicity after CTT via IFN-γ [11]. Thus, NK cells activate ERK signaling in MDSCs via the NKG2D–ligand interaction to increase TACE activity. Increased TACE activity leads to the release of soluble TNF-α, which, in turn, promotes MDSC maturation. Furthermore, mature MDSCs can promote the differentiation of CD4^+^ T cells toward Th1 subsets. In turn, Th1-dominant CD4^+^ T cells can produce IFN-γ to promote MDSC maturation, resulting in feedback regulation to maintain good systemic immune conditions in the host [11]. Our present study further elucidated the relevant mechanism of CTT-induced strong long-term anti-tumor immunity.

## 4. Materials and Methods

### 4.1. Cell Culture and Animal Model

The B16F10 melanoma and 4T1 cell line were provided by Professor Weihai Ying from the Med-X Research Institute, Shanghai Jiao Tong University, and Shanghai First People’s Hospital, Shanghai, China. Cells were cultured in complete Dulbecco’s Modified Eagle Medium (DMEM, GE Healthcare, Logan, UT, USA) with 10% fetal bovine serum (FBS, Gemini Bio-Products, West Sacramento, CA, USA) and 1% penicillin/streptomycin (Hyclone, Logan, UT, USA) in an incubator with 5% CO_2_ at 37 °C.

C57BL/6 and BALB/c female mice at the age of 6~8 weeks were purchased from the Shanghai Slaccas Experimental Animal Co., Ltd. (Shanghai, China). Mice were fed a standard mouse nutritional formula of sterile food and sterile water and housed in isolation cages on a 12 h light/dark cycle. To establish the tumor-bearing mice, the right flanks of C57BL/6 and BALB/c mice were subcutaneously injected with 5 × 10^5^ B16F10 tumor cells and 4 × 10^5^ 4T1 cells. Tumor-bearing mice were randomly assigned to cages and different treatments.

### 4.2. The Cryothermal Therapy (CTT) Procedures

A novel CTT system has been developed in our laboratory. CTT was a treatment system that produce rapidly freezing and radiofrequency heating of the primary tumor. The details of CTT were described in the previous study [49].

Twelve days after tumor inoculation, the size of the tumor reached about 0.2 cm^3^ (the formula V (cm^3^) = width (cm) × length (cm) × height (cm) × π/6). Mice were randomly divided to receive CTT. Tumor-bearing mice were then anesthetized with 1.6% pentobarbital sodium (Sigma-Aldrich, St. Louis, MO, USA) and treated with CTT to eliminate the primary tumor entirely.

### 4.3. NK Cell Depletion In Vivo

To deplete NK cells in vivo, the tumor-bearing mice were injected intraperitoneally (*i.p.*) with anti-NK1.1 monoclonal antibody (clone PK136, Biolegend, San Diego, CA, USA) and IgG2a, κ (clone MOPC-173, Biolegend, San Diego, CA, USA) 250 μg on day 14 after CTT.

### 4.4. Flow Cytometry Analysis

To obtain the single-cell suspension, mice were sacrificed and splenocytes and blood cells were collected. At the same time, to obtain the absolute number of cells, absolute counting beads (424902, Biolegend) were added to the sample. The erythrocytes were then lysed using erythrocyte lysis solution (including 1.0 M of KHCO_3_, 0.1 mM of Na_2_EDTA, and 0.15 M of NH_4_Cl) and resuspended in PBS. The cells were filtered through a 70 μm nylon cell strainer.

Zombie Aqua (BioLegend) was first used to exclude dead cells. For surface markers of cells staining, cells were cultured with different flow cytometry antibodies at 37 °C in a dark place for 15 min.

For intracellular cytokine detection, a cell activation cocktail (Biolegend) was used to stimulate the cells for 4–6 h in an incubator with 5% CO_2_ at 37 °C. The cells were washed in PBS, and surface marking via cell staining was performed before fixation and permeabilization using fixation buffer and intracellular staining permeabilization wash buffer (BioLegend). The cells were then stained with intracellular cytokine antibodies for 20 min.

For transcription factor analysis, a true-nuclear transcription factor buffer set (Biolegend) was used for fixation and permeabilization prior to transcription factor staining. All the antibodies involved are listed in Table S1. LSR Fortessa (BD Bioscience, Franklin Lakes, NJ, USA) and FlowJo (version 10) software was used to collect and to analyze the data, respectively. The flow cytometry gating strategy is shown in Figure S1. Absolute number formula = (number of cells collected × total number of absolute counting beads added)/(number of absolute counting beads collected × spleen weight or blood volume).

### 4.5. 3′ RNA-Seq of MDSCs

Total RNA isolated from MDSCs was extracted using TRIzol reagent (Invitrogen, CA, USA). Then, the purity and concentration of total RNA was analyzed via a NanoDrop 2000 spectrophotometer (Thermo Scientific, Waltham, MA, USA). The extracted total RNA was reverse-transcribed into cDNA via a PrimeScript RT Reagent Kit (TaKaRa, Otsu, Shiga, Japan). An SYBR Green PCR Master Mix (Yeasen, Shanghai, China) and an ABI 7900HT sequence detection system were used for quantitative real-time PCR (qRT-PCR). Table S2 lists all primers used in the experiment.

GO enrichment analysis of differential expression genes was performed separately using R (version 3.2.0), where significantly enriched terms were first screened out and bubble plots of significantly enriched terms were drawn. Principal component analyses (PCAs) were performed using R (version 3.2.0) to assess the biological reproducibility of the samples.

The GSEA software (version 4.2.1) was used for gene set enrichment analysis (GSEA). The GSEA used a predefined set of genes and ranked genes in the order of their degree of differential expression. The predefined gene sets were then examined to see if they were enriched at the top or bottom of the sorted list.

### 4.6. Cell Isolation of CD8^+^ T Cells, NK Cells and MDSCs

Mice from different groups were sacrificed to harvest spleens and separated splenocytes. To obtain CD8^+^ T cells, an EasySep™ Mouse CD8a Positive Selection Kit II (StemCell Technologies, Vancouver, BC, Canada) was used to isolate splenic CD8^+^ T cells. Splenic NK cells were purified via an EasySep™ Mouse NK Cell Isolation Kit (StemCell Technologies) and supplemented with IL-2 (30 IU/mL) for culture in vitro. To obtain splenic MDSCs, APC conjunctive Gr-1 antibody (BioLegend, San Diego, CA, USA) was used to label MDSCs and then isolated using the EasySep™ Release Mouse APC Positive Selection Kit (StemCell Technologies). The isolation of cells was performed according to the manufacturer’s instructions. Cells with a purity of more than 80% were used for experiments.

### 4.7. Coculture Supernatant Preparation and Enzyme Linked Immunosorbent Assay (ELISA)

NK cells, MDSCs alone, and NK cells cocultured with MDSCs were cultured for 24 h in vitro. The supernatant of them was then collected, and TNF-α was detected using the ELISA Kit (Boster Biological Technology, Wuhan, China) according to the manufacturer’s instruction.

### 4.8. In Vitro Tumor Killing Assay

CD8^+^ T cells were isolated from the CTT and the CTT with NK cell depletion groups and cocultured with calcein-AM-labeled B16F10 cells at a ratio of 16:1 in an incubator with 5% CO_2_ at 37 °C [50]. After 6 h, the supernatant was collected in a 96-well black opaque plates to detect the fluorescence level of calcein by enzyme marker (SpectraMax-i3, Molecular Devices, San Jose, CA, USA).

### 4.9. In Vitro MDSCs Phenotype and Function Detection

The suppressive function of MDSCs was defined as the T cell proliferation inhibitory capacity and the phenotypical change of T cells and NK cells. Naive mice were sacrificed to harvest splenocytes, which were then cocultured with MDSCs isolated from different groups. For the detection of T cell proliferation capacity, anti-CD3 antibody (1 μg /mL, clone 145-2C11, Biolegend) was supplied for T cell stimulation, and then cells were cocultured at the indicated ratio for 72 h. The expression of Ki-67 was used to assess the proliferation capacity of CD4^+^ T cells and CD8^+^ T cells using FACS. For evaluating the phenotype of T cells and NK cells, cells were cocultured at a ratio of 1:1 for 24 h. The subset of CD4^+^ T cells and the effector function of CD8^+^ T cells NK cells were analyzed via FACS.

To explore the mechanism of NK cell-mediated MDSCs maturation, MDSCs from the CTT group were isolated and cultured with or without splenic NK cells from the CTT group at a ratio of 1:1 for 24 h in vitro. A transwell chamber (0.4 μm) was used to investigate cell contact. Monoclonal anti-NKG2D antibody (15 μg/mL, clone CX5, Bioxcell), anti-TNF-α (10 μg/mL, clone MP6-XT22, Biolegend), anti-CD40L antibody (15 μg/mL, clone MR-1, Bioxcell), anti-CD86 antibody (15 μg/mL, clone GL-1, Bioxcell), TACE inhibitor TAPI-1 (10 μM, Beyotime), JNK inhibitor SP600125 (10 μM, Beyotime), p38 MAPK inhibitor SB202190 (10 μM, Beyotime), and ERK inhibitor FR180204 (10 μM, Beyotime) were supplied for molecular mechanism clarification.

### 4.10. Western Blotting

Anti-NKG2D (15 μg/mL) antibody was added to coculture the system (containing NK cells and MDSCs) for 3 h; then, MDSCs were isolated from the coculture system. To obtain protein lysates, 1×RIPA lysis buffer containing proteinase inhibitor (Thermo Fisher Scientific, Bremencity, Germany) was used to lyse cells on ice for 30 min. Western blotting was performed as previously described [51]. Anti-p44/42 MAPK (Erk1/2) (Cell Signaling Technology, #4695), anti-Phospho-p44/42 MAPK (Erk1/2) (Cell Signaling Technology, #4370), anti-actin (Santa Cruz, sc-58673), and HRP-labeled secondary antibody (EpiZyme) were used in this study. Relative protein expression levels were quantified using Image J software (version Fiji). The Western blot images shown are representative of three independent experiments.

### 4.11. TACE Activity

MDSCs and NK cells were cocultured with anti-NKG2D (15 μg/mL, clone CX5, Bioxcell) antibody and ERK inhibitor FR180204 (10 μM, Beyotime) for 24 h; then, MDSCs and NK cells were isolated from the coculture system for detection of TACE activity, respectively.

TACE activity was measured using Sensolyte 520 TACE Activity Assay Kit (Anaspec, Fremont, CA, USA) at 490 nm/520 nm, following the manufacturer’s instruction.

### 4.12. Statistical Analysis

All data were presented as mean ± standard deviation (SD). For two groups, two-sided Student’s *T*-test was used to analyze the results. For multiple groups, one-way ANOVA was used to analyze the results. The Kaplan–Meier method and the log-rank test was used to compare the survival rate of mice. All statistical analyses were performed using GraphPad Prism 9.0. A *p*-value lower than 0.05 was considered significant. * *p* < 0.05, ** *p* < 0.01, *** *p* < 0.001, **** *p* < 0.0001.

## 5. Conclusions

In conclusion, the results of the present study revealed that NK cells at the late stage (14 d) after CTT rely on the NKG2D–ligand interaction to increase TACE-cleaved membrane TNF-α activity in MDSCs via ERK signaling, which enhances the soluble TNF-α release from MDSCs, leading to the maturation of MDSCs. Furthermore, mature MDSCs induced by NK cells promote T cell proliferation and the Th1 differentiation of CD4^+^ T cells and enhance the cytotoxicity of CD8^+^ T and NK cells, thereby sustaining CTT-induced long-term survival in mice. Hence, this study not only contributes to a comprehensive understanding of the mechanism by which CTT triggers long-term anti-tumor immunity; it also provides new insights into anti-tumor immunotherapy targeting NK cells and MDSCs.

## Data Availability

The datasets used and/or analyzed during the current study are available from the corresponding author on reasonable request.

## Supplementary Materials

The following supporting information can be downloaded at https://www.mdpi.com/article/10.3390/ijms25105151/s1.
