# Supplementary material for: Natural Killer Cells Reprogram Myeloid-Derived Suppressor Cells to Induce TNF-α Release via NKG2D–Ligand Interaction after Cryo-Thermal Therapy
Source: Int J Mol Sci. 2024 May 9;25(10):5151. doi: 10.3390/ijms25105151 (PMC11121051; doi:10.3390/ijms25105151)
Supplement: Supplementary file 1 [file ijms-25-05151-s001.zip › ijms-2971119-supplementary.pdf]

Supplementary Materials

Supplementary Figures

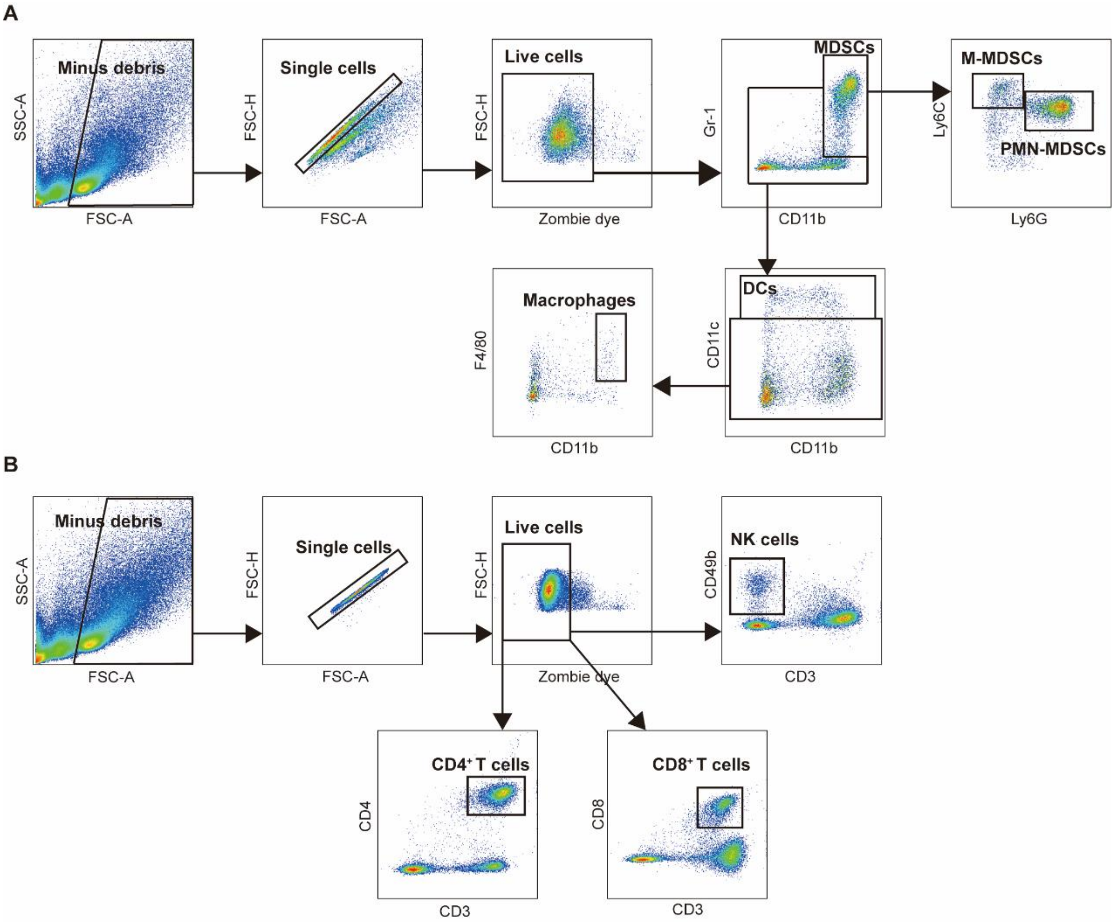

Figure S1. Gating strategy of flow cytometry. (A, B) The gating strategy of myeloid-derived cells (A)and NK cells, T cells (B).

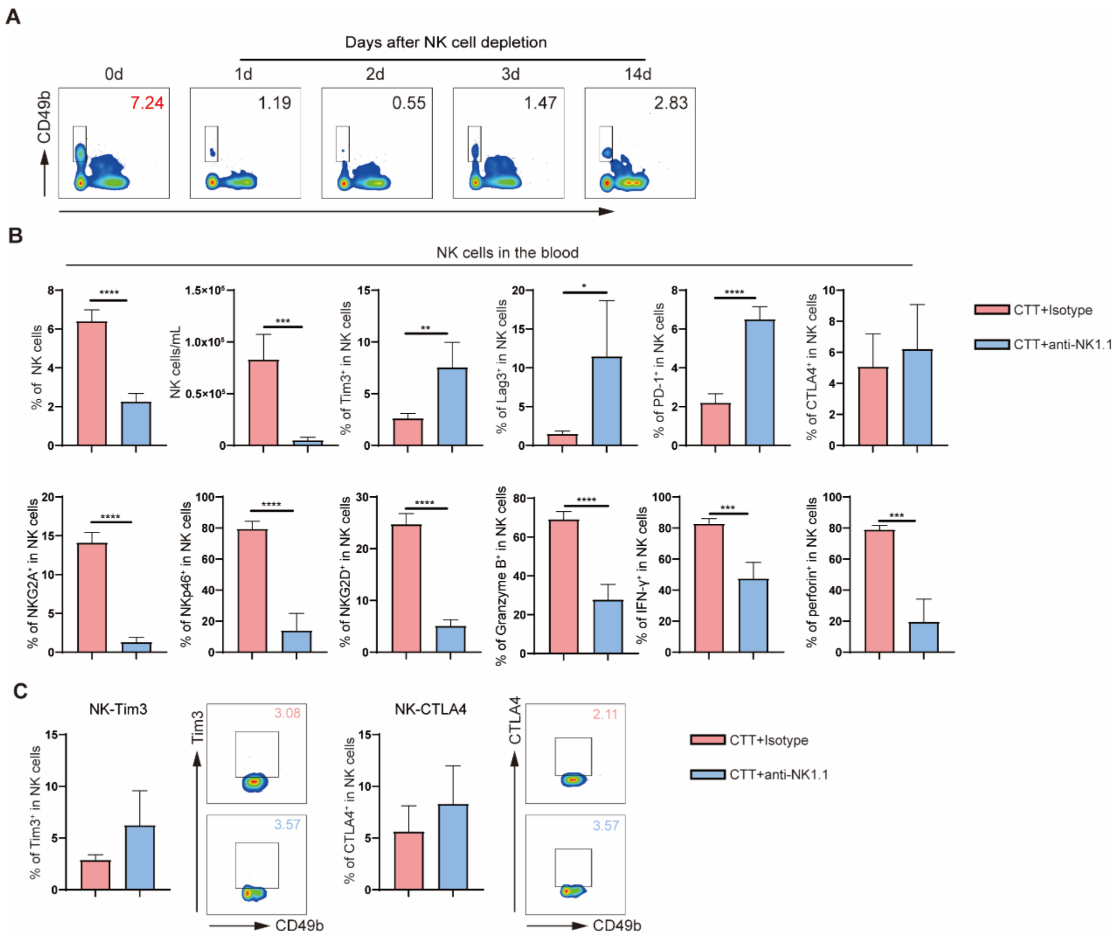

Figure S2. The efficiency of NK cell depletion in the blood. (A) Tumor-bearing mice were treated with CTT on day 12 after inoculation, and 250 μg of anti-NK1.1 was injected i.p. on day 14 after CTT, then the efficiency of NK cell depletion was determined using serial blood draws collected during the two weeks in mice after CTT with NK cell depletion. (B) The percentage, precise number of blood NK cells, as well as the expression of inhibitory molecules (Tim3, Lag3, CTLA4, PD-1), activating and inhibitory receptors (NKG2D, NKp46, NKG2A) and cytotoxic molecules (IFN-γ, Granzyme B, perforin) in blood NK cells. (C) The expression of CTLA4 and Tim3 on educated NK cells by CTT and naive NK cells in the spleen. All data were shown as mean ± SD. n=4 for each group. **P* < 0.05, ***P* < 0.01, ****P* < 0.001, *****P* < 0.0001. Data for graphs were calculated by using two-sided Student's T-test.

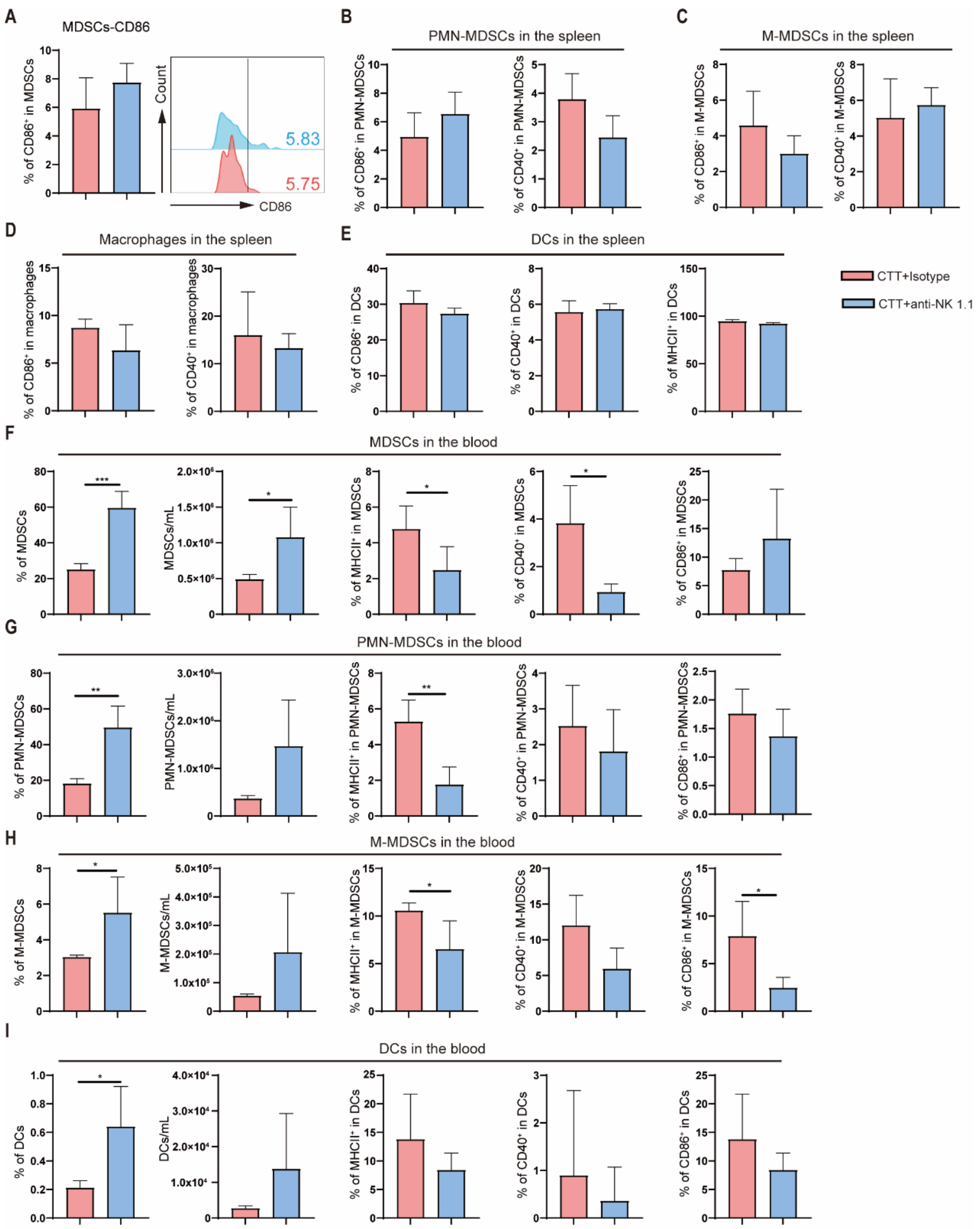

Figure S3. The amount and phenotypes of MDSCs and DCs in the blood. (A) The expression of CD86 on splenic MDSCs. (B-D) The expression of CD86 and CD40 on splenic PMN-MDSCs (B) M-MDSCs (C) and macrophages (D). (E) The expression of CD86, CD40 and MHC-II on splenic DCs. (F-I) The percentage, absolute number, MHC II, CD40 as well as CD86 expression of MDSCs (F) PMN-MDSCs (G) M-MDSCs (H) and DCs (I) in the blood. All data were shown as mean ± SD. n=4 for each group. *P < 0.05, **P < 0.01, ***P < 0.001. Data for graphs were calculated by using two-sided Student's T-test.

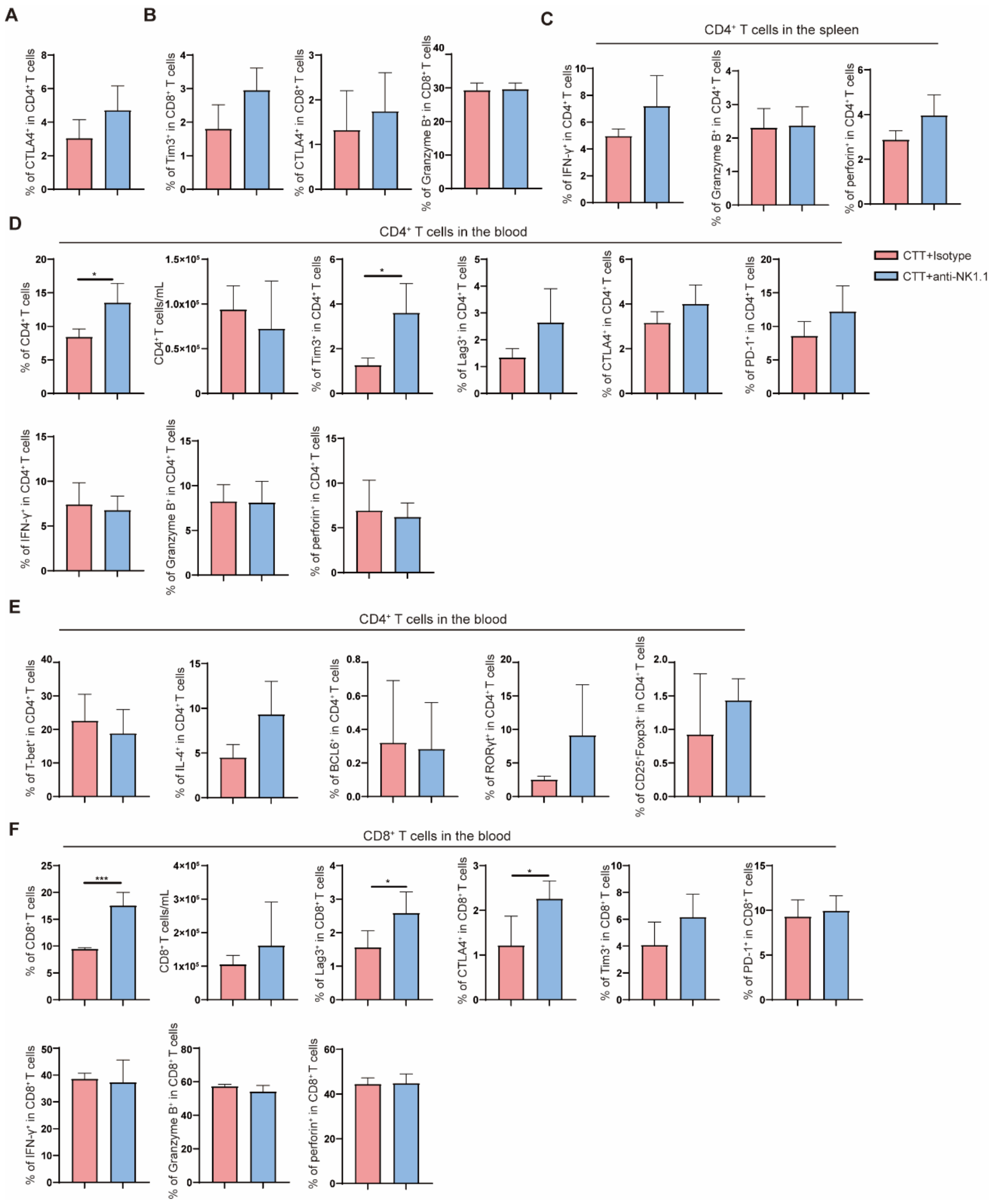

Figure S4. The phenotypical changes of CD4$^+$ T cells, CD8$^+$ T cells and NK cells after CTT with NK cell depletion. (A) The expression of CTLA4 on splenic CD4$^+$ T cells. (B) The expression of Tim3, CTLA4 and Granzyme B in splenic CD8$^+$ T cells. (C) The expression of IFN-γ, Granzyme B, perforin in splenic CD4$^+$ T cells. (D) The percentage, precise number of blood CD4$^+$ T cells, as well as the expression of inhibitory molecules (Tim3, Lag3, CTLA4, PD-1), cytotoxic molecules (IFN-γ, Granzyme B, perforin) in blood CD4$^+$ T cells. (E) The subsets of CD4$^+$ T cell in the blood. (F) The percentage, precise number of CD8$^+$ T cells, as well as the expression of inhibitory molecules (Tim3, Lag3, CTLA4, PD-1) and cytotoxic cytokines (IFN-γ, Granzyme B, perforin) in CD8$^+$ T cells in the blood. All data were shown as mean ± SD. n=4 for each group. *$P < 0.05$, ***$P < 0.001$. Data for graphs were calculated by using two-sided Student's T-test.

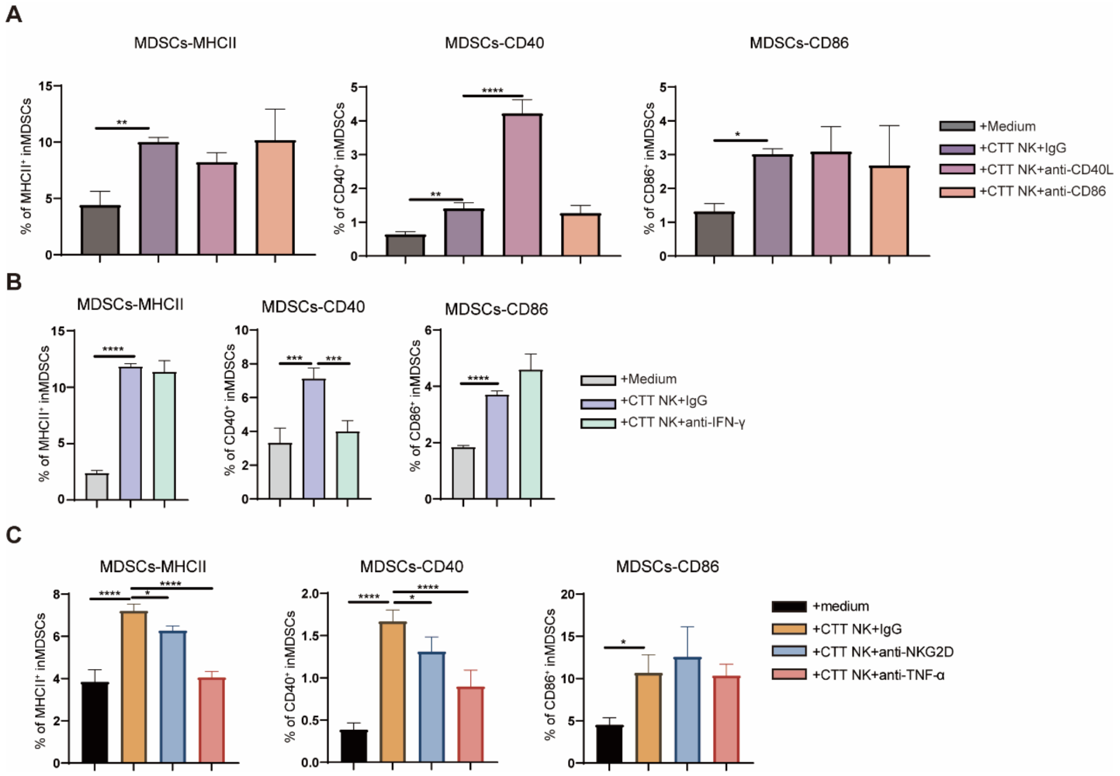

Figure S5. NK cells regulated MDSC maturation at the late stage after CTT. (A, B) MDSCs and NK cells were cocultured with the addition of anti-CD40L, anti-CD86 (A) and anti-IFN-γ (B) antibodies. The expression of MHC II, CD86 and CD40 on MDSCs were detected by flow cytometry. (C) In 4T1 model, MDSCs and NK cells were cocultured with the addition of anti-TNF-α antibodies. The expression of MHC II, CD86 and CD40 on MDSCs were detected by flow cytometry. All data were shown as mean ± SD. n=4 for each group. *P < 0.05, **P < 0.01, ***P < 0.001, ****P < 0.0001. Data were analyzed using one-way ANOVA.

Table S1. Flow cytometry antibodies used in this study

Antibodies	Clone	Source	Cat#
Pacific Blue anti-mouse CD11b	M1/70	BioLegend	101224
APC anti-mouse Gr-1	RB6-8C5	BioLegend	108412
FITC anti-mouse Ly-6C	HK1.4	BioLegend	128006
PE/Cyanine7 anti-mouse Ly-6G	1A8	BioLegend	127618
PE anti-mouse CD11c	N418	BioLegend	117306
Brilliant Violet 711™ anti-mouse F4/80	BM8	BioLegend	123147
APC/Cyanine7 anti-mouse CD86	GL-1	BioLegend	105030
PerCP/Cyanine5.5 anti-mouse I-A/I-E	M5/114.15.2	BioLegend	107626
BUV395 Anti-Mouse CD40	3/23	BD Biosciences	745697
PerCP/Cyanine5.5 anti-mouse CD3ε	145-2C11	BioLegend	100328
FITC anti-mouse CD3ε	145-2C11	BioLegend	100306
APC/Cyanine7 anti-mouse CD3ε	145-2C11	BioLegend	100330
PE/Cyanine7 anti-mouse CD4	RM4-5	BD Biosciences	552775
APC anti-mouse CD4	RM4-5	BD Biosciences	561091
Pacific Blue anti-mouse CD8a	53-6.7	BioLegend	100725
Alexa Fluor® 700 anti-mouse CD8a	53-6.7	BioLegend	100730
PerCP/Cyanine5.5 anti-mouse CD49b	DX5	BioLegend	108916
PE/Cyanine7 anti-mouse CD25	3C7	BioLegend	101916
PE anti-mouse Foxp3	MF-14	BioLegend	126404
Brilliant Violet 605™ anti-T-bet	4B10	BioLegend	644817
Brilliant Violet 421™ anti-mouse IL-4	11B11	BioLegend	504120
BV421 Mouse Anti-Bcl-6	K112-91	Biosciences	563363
ROR-γt	Q31-378	Biosciences	564723
Brilliant Violet 605™ anti-mouse IFN-γ	XMG1.2	BioLegend	505840
Alexa Fluor® 647 anti-human/mouse Granzyme B	GB11	BioLegend	515406
PE anti-mouse Perforin	S16009A	BioLegend	154306
Brilliant Violet 711™ anti-mouse LAG-3	C9B7W	BioLegend	125243
PE anti-mouse PD-1	29F.1A12	BioLegend	135206
Brilliant Violet 421 anti-mouse Tim-3	5d12	Biosciences	119723
Brilliant Violet 421 anti-mouse CTLA-4	UC10-4B9	BioLegend	106312
APC anti-mouse NKG2A	16A11	BioLegend	142808
Brilliant Violet 421 anti-mouse NKp46	29AL4	BioLegend	137611
PE anti-mouse NKG2D	A10	BioLegend	115606

BUV395 anti-mouse Ki-67	B56	Biosciences	564071
FITC anti-mouse TNF-α	MP6-XT22	BioLegend	506303

Table S2. Primer sequences of genes in this study

Name	Primer Sequence (5´-3´)
IL-1β-F	ACAGCAGCACATCAACAAGAG
IL-1β-R	ATGGGAACGTCACACACCAG
IL-7-F	TTCCTCCACTGATCCTTGTTCT
IL7-R	AGCAGCTTCCTTTGTATCATCAC
TGF-β-F	CTCCCGTGGCTTCTAGTGC
TGF-β-R	GCCTTAGTTTGGACAGGATCTG
IL-15-F	AGAGGCCAACTGGATAGATGT
IL-15-R	AGAGCACGTTTCTTACTGTTTCA
MHC II-F	AGCCCCATCACTGTGGAGT
MHC II-R	GATGCCGCTCAACATCTTGC
CD86-F	GAGCTGGTAGTATTTTGGCAGG
CD86-R	GGCCCAGGTACTTGGCATT
CXCL10-F	CCAAGTGCTGCCGTCATTTTC
CXCL10-R	GGCTCGCAGGGATGATTTCAA
CXCL9-F	GGAGTTCGAGGAACCCTAGTG
CXCL9-R	GGGATTTGTAGTGGATCGTGC
TNF-α-F	TTCTGTCTACTGAACTTCGGGGTGATCGGTCC
TNF-α-R	GTATGAGATAGCAAATCGGCTGACGGTGTGGG
IL-6-F	GACAAAGCCAGAGTCCTTCAGAGAGATACAG
IL-6-R	TTGGATGGTCTTGGTCCTTAGCCAC
CD40-F	TGTCATCTGTGAAAAGGTGGTC
CD40-R	ACTGGAGCAGCGGTGTTATG
